# Etiology and management of hyperimmunoglobulinemia E syndrome

**DOI:** 10.4103/0301-4738.62659

**Published:** 2010

**Authors:** L I Gonzalez-Granado

**Affiliations:** Immunodeficiencies Unit, Hospital 12 octubre, 28041 Madrid, Spain

Dear Editor

I read with interest the article ‘Ophthalmic complications including retinal detachment in hyperimmunoglobulinemia E (Job's) syndrome: Case report and review of literature’ by Arora *et al*.[1] I am grateful for their contribution to ocular manifestations of hyperimmunoglobulinemia E (hyper-IgE) syndrome (formerly Job's syndrome). However, I would like to make some comments.

First, the authors state that the origin of this disease is unknown. Fortunately, the etiology of hyper-IgE syndrome, in most cases, has been discovered in the last three years. In 2006 Tyk-2 gene mutations were acknowledged as the underlying cause in patients with an autosomal recessive inheritance.[2] Afterwards, Holland *et al*. reported that autosomal dominant HIES, the most common disease in this group (almost two-thirds), results from STAT3 mutations.[3]

Secondly, the maintenance treatment is based on immunoglobulin replacement therapy or antibiotic (anti-staphylococcal) prophylaxis. However, azithromycin is not a suitable drug for *Staphylococcus aureus* (neither as prophylaxis nor as treatment). First-generation cephalosporins or cotrimoxazole should be the “first line” drugs. Unfortunately, bone marrow transplantation is not effective.[4]

Finally, the authors speculate vigorous rubbing of eyes due to intense itching to be a probable cause of retinal detachment. There are no other cases reported in the literature with this association. Moreover, pathogenesis in autosomal dominant cases involves Th17 cells (a subgroup of regulatory peripheral T cells, the development of which is interrupted in hyper-IgE syndrome). Atopic dermatitis needs to be ruled out whenever hyper-IgE syndrome is considered. In this scenario, Grimbacher score may help to guide gene sequencing in order to confirm the clinical suspicion.[5] Fortunately, most cases suspected to be Job's syndrome are finally diagnosed as only atopic dermatitis.
